# Recent research progresses of bioengineered biliary stents

**DOI:** 10.1016/j.mtbio.2024.101290

**Published:** 2024-10-05

**Authors:** Jianing Yan, Zhichao Ye, Xiaofeng Wang, Danyang Zhong, Ziyuan Wang, Tingting Yan, Tianyu Li, Yuyang Yuan, Yu Liu, Yifan Wang, Xiujun Cai

**Affiliations:** a Department of General Surgery, Sir Run Run Shaw Hospital Affiliated to School of Medicine, Zhejiang University, Hangzhou, 310016, China; b National Engineering Research Center of Innovation and Application of Minimally Invasive Instruments, Sir Run Run Shaw Hospital, School of Medicine, Zhejiang University, Hangzhou, 310028, China; c Department of Translational Medicine & Clinical Research, Sir Run Run Shaw Hospital, School of Medicine, Zhejiang University, Hangzhou, 310028, China; d Department of Plastic Surgery, Sir Run Run Shaw Hospital, Zhejiang University School of Medicine, Hangzhou, 310016, Zhejiang Province, China

**Keywords:** Biodegradable stents, Bioengineering materials, Biliary cell sources, Stent fabrication, 3D bio-printing

## Abstract

Bile duct lesion, including benign (eg. occlusion, cholelithiasis, dilatation, malformation) and malignant (cholangiocarcinoma) diseases, is a frequently encountered challenge in hepatobiliary diseases, which can be repaired by interventional or surgical procedures. A viable cure for bile duct lesions is implantation with biliary stents. Despite the placement achieved by current clinical biliary stents, the creation of functional and readily transplantable biliary stents remains a formidable obstacle. Excellent biocompatibility, stable mechanics, and absorbability are just a few benefits of using bioengineered biliary stents, which can also support and repair damaged bile ducts that drain bile. Additionally, cell sources & organoids derived from the biliary system that are loaded onto scaffolds can encourage bile duct regeneration. Therefore, the implantation of bioengineered biliary stent is considered as an ideal treatment for bile duct lesion, holding a broad potential for clinical applications in future. In this review, we look back on the development of conventional biliary stents, biodegradable biliary stents, and bioengineered biliary stents, highlighting the crucial elements of bioengineered biliary stents in promoting bile duct regeneration. After providing an overview of the various types of cell sources & organoids and fabrication methods utilized for the bioengineering process, we present the in vitro and in vivo applications of bioengineered biliary ducts, along with the latest advances in this exciting field. Finally, we also emphasize the ongoing challenges and future development of bioengineered biliary stents.

## Graphical abstract

**Figure:**
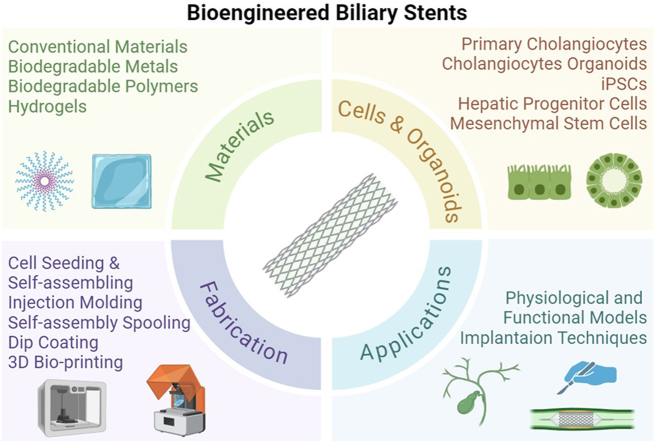

## 1 Introduction

Biliary system consists of intrahepatic and extrahepatic bile duct, and transfers bile, which is secreted by hepatocytes, into digestive tract. Under normal condition, the liver produces approximately 600–1200 ml of bile flow per day [1]. Bile flow rates can vary depending on various factors, including diet and liver function. In pathological conditions, such as biliary obstruction or cholestasis, the flow rate of bile can be significantly reduced, leading to liver function injured and potentially causing jaundice or other complications.

In human, the total length of the biliary system is nearly 2 km [2]. Beginning from bile canaliculus, bile ducts gradually converge to small bile duct, large bile duct, hepatic duct, common hepatic duct and common bile duct (CBD) [2]. The CBD is 4–8 cm in length with a diameter of 0.6–0.8 cm. The CBD usually joins the main pancreatic duct and opens at the duodenal papilla where the Oddi's sphincter is formed, controlling bile flow and preventing reflux. The outer layer of the CBD is the serosa layer, while the middle layer is composed of connective tissue and muscle fibres, and the inner layer is the mucosal layer. The blood supply to the CBD mainly comes from the anonymous veins that originate from the superior pancreaticoduodenal artery and the cystic artery, which nourish the CBD through a vascular network [3].

Biliary system diseases include occlusion, cholelithiasis, dilatation, malformation, and cholangiocarcinoma, which can result in varying degrees of bile duct lesion. According to different types of lesions, bile duct lesion can be treated by surgical and interventional operations [[4], [5], [6], [7]]. Surgical treatment is the therapy with the most definite effect of severe bile duct lesion. Intrahepatic bile duct diseases are often diffused, and treated by partial hepatectomy or liver transplantation [8,9]. And in extrahepatic bile duct lesion, cholangiojejunostomy is the most common performed surgical method [10,11] to reconstructs biliary-enteric passage which is adequate to various kinds of bile duct lesion. However, cholangiojejunostomy would change the physiological drainage of bile, influence homeostasis of hormone in digestive tract and sacrifice the function of Oddi's sphincter. This is accompanied by a number of complications, such as anastomotic ulcer, duodenal ulcer, reflux cholangitis, stricture, even if cholangiocarcinoma, which are commonly occurred after cholangiojejunostomy [12].

Biliary stents procedure are an ideal therapeutic method with great potential [13]. Biliary stents are typically inserted endoscopically or through percutaneous and surgical methods, aiming to relieve obstruction in biliary tract caused by conditions such as gallstones, tumours, or inflammation. As avoiding the damage of native construction of Vater ampulla, stents can serve as minimally invasive procedure to alleviate biliary strictures. Nowadays, implanted biliary stents are commonly made of plastic and metal. However, the clinical use of plastic or metal biliary stents is highly possible to cause a range of significant complications, such as cholecystitis, pancreatitis, restenosis and corrosion-related problems [14], which lead to additional damage to patients [[15], [16], [17]]. Plastic stents must be replaced 3–6 months after implantation, and patients who still have biliary strictures after several times of stents implantation require surgical treatment [15]. Metal stents have a higher incidence of cholangitis and cholelithiasis, and they are more probably to embedding into the bile duct wall, making later surgical repair more difficult and the prognosis worse. A meta-analysis study reveals that the incidence of pancreatitis was higher after deployment of metal stents compared to plastic ones [18]. As a result, metal stents should be avoided whenever possible to treat bile duct lesion [19]. However, both of them shared overall adverse events included pancreatitis, cholangitis, perforation, haemorrhage, abdominal pain, cholecystitis, infection, and stent dysfunction such as stent occlusion, migration unravelling, and non-removability. Therefore, it is important to select a biocompatible biomaterial with good mechanical properties and less immune rejection to develop artificial biliary stents suitable for implantation in the body. Under ideal conditions, biomaterials can temporarily or permanently replace the function of the impaired tissue or organ, supporting the reconstruction of the structure and restoration of physiological function. Bioengineering artificial biliary stents, which are made of well-compatible biomaterials and with laden seeding cells, can provide support of cell adhesion, migration and proliferation at the defect site of bile duct, promoting biliary regeneration and finally repairing bile injury with the most optimal outcome [20,21]. Furthermore, scaffold-free tissue-engineering biliary stents can be assembled by bio fabricating technology, which is closer to the formation of natural bile duct and accomplish to a perfect repairing [22].

**Fig. 1:**
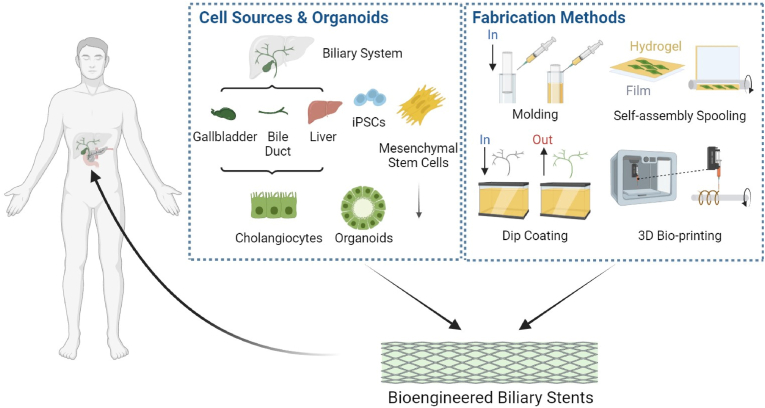
Cell sources & organoids, fabrication methods and applications of bioengineered biliary stents. Created with BioRender.com. iPSCs, Induced Pluripotent Stem Cells.

In the past decades, several great achievements in bioengineered biliary stents have been obtained. In this review, we provide a comprehensive overview of the development of conventional biliary stents, biodegradable biliary stents, and bioengineered biliary stents, highlighting the crucial elements of bioengineered biliary stents in promoting bile duct regeneration. Additionally, we investigate the various types of seeding cells and fabrication methods utilized for the bioengineering process (Fig. 1). Finally, we summarize the in vitro and in vivo characterization methods of bioengineered biliary ducts, along with the latest advances in this exciting field.

## 2 Materials for stent fabrication

### 2.1 Conventional materials

The application of biliary stents is a critical step in therapeutic development of bile duct lesion [23]. Conventional biliary stents, mainly including plastic and metal stents, are widely used for several advantages. They offer a less traumatic method for resolving biliary lesion, which is generally considered safe with a relative low rate of complications. Meanwhile, conventional biliary stents are designed to conform to the physiological and anatomical compatibility of the biliary tract, which helps in maintaining proper bile flow. They can serve as a preferred palliative treatment for biliary obstructions caused by unresectable pancreaticobiliary tumours, providing relief and improving the quality of life for patients who are unsuitable for more aggressive treatments. And conventional stents, with plastic stents in particular, are relatively inexpensive and easily accessible, making them a common choices especially for patients with a short life expectancy. Moreover, conventional stents offer long patency times, which resists compression and maintains structural integrity.

**Fig. 2:**
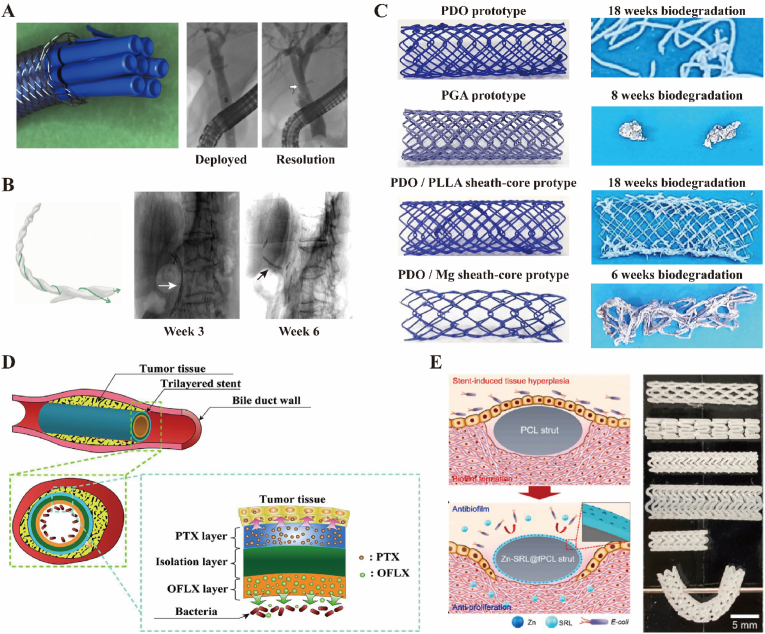
Biliary stents with different materials, shapes, biodegradability and functions. (A) A 10 mm diameter Fully Covered Self-Expandable Metal Stent corresponds to seven 10 Fr plastic stents [32]. Copyright © 2019, Andrea Tringali et al. (B) A biodegradable biliary stent with the helicoidal shape that allows for bile flow from the inside channel and along the outer stent surface [49]. Copyright © 2020 by the American Society for Gastrointestinal Endoscopy. (C) The biodegradation time of biodegradable polymer biliary stents and conventional biliary stents [50]. Copyright © 2020 Japan Gastroenterological Endoscopy Society. (D) A tri-layered film for biliary stents with antibiotic coatings and antitumour coatings [51]. Copyright © 2021 Elsevier B.V. (E) A polymer biodegradable biliary stent with nanoengineered surfaces that prevents hyperplasia and bacterial responses [52]. Copyright © 2024, KeAi Communications Co. Ltd.

However, conventional biliary stents are usually non-biodegradable [24,25]. The clinical usage of plastic or metal stents can lead to a series of significant complications, such as stent obstruction, occlusion, impaction, cholangiolithiasis, and biliary tract infection [16,17]. Relatively, plastic stents are more prone to trigger infectious complications, compared to metal stents [26].Both plastic and metal stents have their share of disadvantage that sometimes they are difficult to remove due to tissue over-growth [27]. To avoid early stent occlusion, multiple plastic stents and self-expanding metal stent holds considerable promise for maximum effect [28,29]. What's more, with the development of covered self-expanding metal stents, generally covered by a polytetrafluoroethylene layer, biliary stent with higher expansion force and larger diameter has reduced the probability of stents incarceration after implantation [30,31]. As reported, a single fully-covered self-expanding metal stent expands to a diameter similar to that of the largest bundle of multiple plastic stents after approximately 1 year of multi-procedure treatments (Fig. 2A) [32]. Drug-eluting stents offers additional benefits. For example, a novel paclitaxel-eluting biliary metal stent incorporating sodium caprate reduced tumour volume [33] and novel antimicrobial coatings can prevent cholangitis [34]. Indeed, they still require frequent endoscopic stent exchanges or even to be removed by a secondary surgery, which brings consequent burden to patient [15,35,36].

### 2.2 Biodegradable metals

Biodegradable metals have become possible materials that are widely utilized in fabrication of temporary implanted devices for cardiovascular [37], cerebrovascular [38] and orthopaedic [39,40] diseases. Biodegradable metals mainly include magnesium-, zinc-, and iron-based alloys, anticipating to decompose gradually after rehabilitation [41]. Compared to polymeric materials, biodegradable metals have stronger mechanical strength and function better as implants and stents.

Recent advancements in Mg-based stents present promising results. Zan et al. developed COF-based nanocomposite coatings for Mg alloy stents, enhancing degradation resistance and biocompatibility [42]. Peng et al. systematically investigated the degradation behaviour of HP-Mg (HPM) and Mg-2 wt.%Zn (MZ2), after which they found high-purity Mg wires inhibit gallbladder cancer and effectively drain bile [43]. Liu et al. demonstrated Mg-based AZ31 stents retain significant volume and reduce inflammation in rabbit bile ducts, noting non-uniform corrosion [44]. Building on these advancements, the Unity-B biliary stent, a CE-marked biodegradable metal stent, offers excellent biocompatibility, appropriate degradation rates, and robust mechanical properties, thereby reducing long-term complications and significantly improving patient outcomes [45]. Moreover, in clinical practice, a case report explores the use of a novel biliary biodegradable stent (BEBS) for managing post cholecystectomy perihilar benign biliary strictures, highlighting its continuous dilation advantage and potential for stent-in-stent (Y-shaped) configurations, beneficial for complex or indeterminate biliary strictures [46]. Unlike vascular stents, which can be treated with surface heparinization to prevent thrombosis and obstruction, biliary stents are surrounded by the environments where bile acids, cholesterol, bilirubin, and potential retrograde infection by operation can easily lead to the formation of stones. The metal ions from degraded stents may bind with bile acids, or chelate with cholesterol and bilirubin, contributing to form crystal nuclei of stones and leading to secondary cholangitis and recurrent stenosis [47,48]. Therefore, research on bioengineered biliary stents tends to use biopolymer materials more frequently.

### 2.3 Biodegradable polymers

Although conventional biliary stents allow clinicians to achieve transient maintenance of bile duct patency, they still suffer from the above limitations, which mainly stem from the substrate materials. Tissue engineering biliary stents are considered as the next generation of biliary stents. The comparisons between different materials for tissue engineering biliary stent fabrication have been demonstrated in Table 1. Materials with favourable mechanical properties, excellent biocompatibility and complete biodegradability is required for fabrication of artificial biliary stent [53]. Benefiting from the advances in biodegradable polymer materials, such as poly lactic-co-glycolic acid (PLGA), polyglycolide (PGA), polydioxanone (PDO), poly-L-lactic acid (PLLA) and polycaprolactone (PCL), options have expanded from conventional matters to a wider variety of materials. The degradation modes of biomaterials in vivo include hydrolysis, oxidative degradation and enzymatic hydrolysis [54]. Controlling the degradation rate of biodegradable materials is critical for their biomedical applications. For example, Yuan Feng et al. prepared three-dimensional porous polyurethane-hyaluronic acid (PUHA) hybrid hydrogel scaffolds and the appropriate degradation rate of PUHA hydrogel scaffolds can be adjusted by the content of HA for specific applications [55]. The biodegradable polymer biliary stents can reduce the burden of secondary surgery on patients [45]. For instance, Siiki et al. first reported the clinical experience of endoscopically inserted polydioxanone biodegradable biliary stent in patients with benign common bile duct stricture [56]. Furthermore, they registered a clinical trial (NCT02353286) and verified the advantages of PDO-based self-expanding biodegradable stent in avoiding of repeated endoscopy for stent removal. Though most implantation of biodegradable stents succeeded, nearly 17 % patients developed stricture relapse and one-third of patients underwent acute cholangitis [57]. Similarly, Mauri et al. performed percutaneous placement of PDO-based biodegradable stent. However, they also reported nearly 20 % of stricture recurrence and higher rate of cholangitis and biliary stone [58]. Therefore, although we have witnessed the development of biodegradable polymer biliary stents for primary attempt in medical practice, they are still in the early stages of clinical adoption, requiring evaluation of feasibility and effectiveness.

**Table 1:** Advantages and disadvantages of different materials for tissue engineering biliary stents fabrication.

Materials	Advantages	Disadvantages	Ref
Magnesium-based Alloys	High biocompatibility;	Rapid degradation rate leading to premature loss of mechanical integrity	[41]
Zinc-based Alloys	1. Moderate degradation rate; 2. High biocompatibility;	1. Limited mechanical strength 2. Potential for toxic by-products	[41]
Iron-based Alloys	1. Slow and controlled degradation rate 2. High mechanical strength	1. Potential for localized iron overload 2. Possible inflammatory response	[41]
Polyurethane-hyaluronic acid (PUHA)	1.High water retention for moisture; 2.Recoverable compression; 3.Covalent crosslinking for stability; 4.Simple design for immune modulation;	1. Ignoring immune modulation; 2. Limited mechanisms of modulating the polarization of macrophages;	[55]
Polydioxanone (PDO)	1.High biocompatibility; 2.Shape memory and mechanical properties; 3.Modulation of degradation;	1. Processing control challenges; 2. Relatively high costs;	[[56], [57], [58]]
Poly(lactic acid) (PLA)	1.High biocompatibility; 2.Advanced mechanical properties and sustainability; 3.Controlled degradation;	1. Brittleness; 2. Acidic degradation products; 3. Limited cell adhesion; 4. Limited Oxygen Permeability;	[51]
Poly lactic-co-glycolic acid (PLGA)	1. Tunable Degradation Rate; 2. High biocompatibility; 3. Advanced mechanical properties and sustainability; 4. Regulatory acceptance;	1. Acidic degradation products; 2. Limited cell adhesion;	[91]
Polycaprolactone (PCL)	1. Tunable Degradation Rate; 2. High biocompatibility; 3. Advanced mechanical properties and sustainability; 4. Fabrication flexibility;	1. Slow degradation Rate; 2. Low tensile strength; 3. Limited cell adhesion;	[52]
decellularized extracellular matrix (dECM)	1. High biocompatibility; 2. Preservation of native ECM; 3. Promotion of cell function;	1. Immunogenicity; 2. Low material property;	[68,69]
Methacrylate gelatin (GelMA)	1. High biocompatibility; 2. Preservation of native gelatin; 3. Promotion of cell versatility;	1. Degradation control; 2. Low material property;	[92]
Sodium alginate	1. High biocompatibility; 2. High viscosity in water; 3. pH sensitivity;	1. Limited solubility; 2. Excessive sensitivity to environmental conditions 3. Over reactivity with divalent cations	[75]
Hyaluronan	1. Hydrophilicity; 2. High biocompatibility; 3. Visocoelastic properties	1. Interaction with other substances 2. Short effective duration	[82]

Moreover, a suitable structure capable of facilitating bile flow is necessary for advanced biliary stents. For example, Anderloni et al. designed a helicoidally shaped biodegradable biliary stent (Archimedes stent; Amg International GmbH, Winsen, Germany), also based on PDO, to favour bile flow while maintaining duct unobstructed (Fig. 2B) [49]. These biliary stent could self-degrade in 6 weeks after deployment. Besides, mechanical properties and degradation rates of biliary stents are different according to multiple structures and materials. Thus, it is crucial to evaluate degradation process and time of various biodegradable polymer biliary stents to choose appropriate types on a patient-by-patient basis to provide a clinically effective option. Kwon et al. simultaneously evaluated the mechanical properties and degradation time of several biodegradable polymer materials, including PDO prototype, PGA prototype, PDO/PLLA sheath-core prototype and PDO/magnesium (Mg) sheath-core prototype (Fig. 2C) [50].

To further functionalize the biliary stents, researchers add various coatings to the stent, including antibiotic and antitumour coatings, for various requirements of different clinical cases. For instance, Liu et al. designed and prepared a tri-layered film for biliary stents (Fig. 2D) [51]. The antitumour poly (lactic acid) (PLA) layer loaded with chemicals, releasing paclitaxel (PTX) to cure tumour. The antibacterial PLA layer loaded with ofloxacin (OFLX), avoiding the formation of biofilms on the surface of stent. The middle PLA isolation layer separated the PTX layer from the OFLX layer. Lee et al. fabricated functionalized polymer biodegradable biliary stents with nanoengineered surfaces which were created on a sirolimus (SRL)-coated functionalized ploy(ε-carprolactone) (fPCL) stent using Zn ion sputtering-based plasma. With these nanoengineerd surface, Zn-SRL@fPCL stent sufficiently inhibited bacterial colonization and fibroblast responses (Fig. 2E) [52].

### 2.4 Hydrogels

Significantly, the superior mechanical properties and excellent biocompatibility of the biliary stent improve the effectivity of bile drainage [59]. Despite many advances in fabrication of biodegradable polymer scaffolds, highly biomimetic biliary stents still leave us with difficulty to achieve. Biliary stents, which are currently used in clinical, simply mimic normal bile duct morphology. Their limitations in promoting biliary tree regeneration, bile duct vascularization and biliary cells migration still remain. Hydrogel materials can provide micro-environmental support for cell growth and migration, which is crucial for maintaining specific three-dimensional morphology and physiological function over the long term. The ideal hydrogel materials should simulate the extracellular matrix (ECM) in tissues, degrade steadily, and be replaced by the extracellular matrix synthesized by cells [60]. Additionally, the material should have physical and chemical properties similar to those of the ECM [61,62]. The ECM is a key component that maintains the shape and function of organs in the human body. Therefore, in order to simulate the ECM more accurately, relevant studies are focused on artificially modifying biomaterials to improve their biocompatibility, enrich their growth factors, and make them more suitable for cell growth and function.

**Fig. 3:**
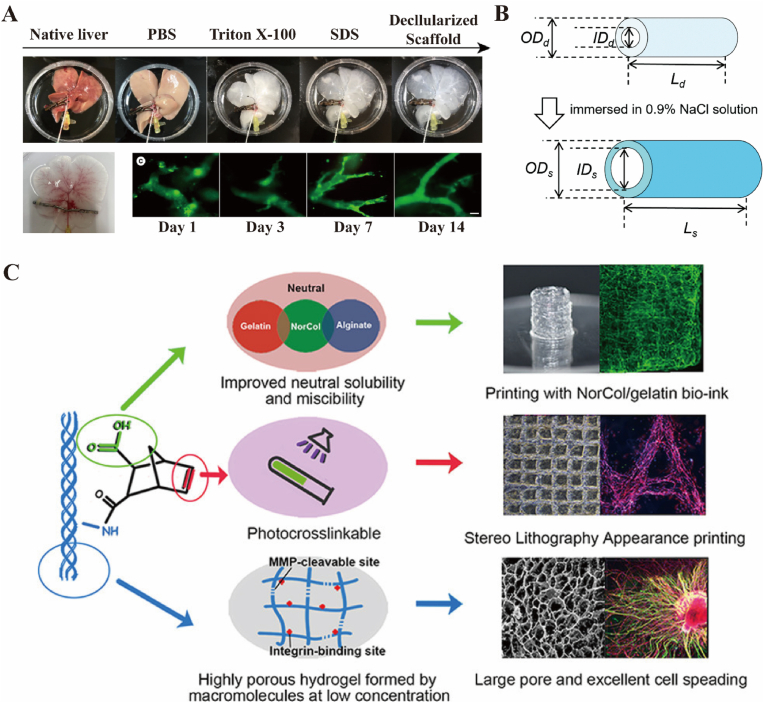
Bioengineering materials. (A) Decellularization in liver and construction of functional ductal organoids [65]. Copyright © 2023, KeAi Communications Co. Ltd. (B) The swelling behaviour of hydrogel stent [74]. Reproduced from Ref. 47 with permission from the Royal Society of Chemistry. (C) A synthesized norbornene-functionalized neutral soluble collagen (NorCol) enabled for temperature-based, ion-based, and photo-based bio-printing [90]. Copyright © 2021, American Chemical Society.

Currently, decellularization of solid organs can preserve the ECM and even many growth factors related to regeneration. Mayorca-Guiliani et al. developed an ECM-specialized method of decellularization in liver [63], and Tomofuji et al. successfully reconstructed intrahepatic bile ducts in a decellularized liver acquired by this method [64]. Moreover, Chen et al. developed functional duct organoids (FDOs) with biliary tree networks in decellularized liver scaffolds. Theses FDOs maintained specific biomarkers, basal-apical polarity and metabolic expression [65] (Fig. 3A), indicating superb compatibility of decellualarized liver for biliary organoids construction. Therefore, decellularized extracellular matrix (dECM) is a promising biomaterial in the field of regenerative medicine due to their unique biological activities and excellent biocompatibility. dECM hydrogels can absorb and preserve a certain amount of water, simulating the water environment of human tissues, facilitating nutrient exchange, oxygen supply, and waste removal, and making them ideal for tissue engineering applications [66]. Rosen et al. utilized decellularized porcine small intestinal submucosa (SIS) as a scaffold for biliary tract regeneration due to its high biocompatibility, biodegradability, and predominantly collagenous composition. Following implantation into a canine model of biliary tract injury, autologous bile duct epithelium and collagen replaced the SIS graft after three months [67]. Similarly, Cheng et al. employed acellular ureter as a biliary stent to repair a damaged CBD, whereas Li et al. utilized a basic fibroblast growth factor (bFGF) collagen-loaded decellularized scaffold for bile duct repairing. The incorporation of bFGF into the collagen binding domain accelerated the generation of blood vessels and enhanced tissue regeneration, resulting in reduced complications, inflammation, and improved biliary epithelium and gland regeneration with regular tissue morphology following implantation [68].

Synthetic hydrogels share similar characteristics and can also preserve a certain amount of water through a hydrophobic bond crosslinking network [69]. Hydrophobic hydrogels have their unique advantages in the preparation of scaffolds owes to excellent mechanical properties [70]. The hydrophobic interactions not only increase the resistance of the hydrogel to water loss and evaporation, effectively preserving water within the gel, but also reduces the mobility of water molecules within the hydrogel network [71,72]. Compared with dECM, the composition of synthetic hydrogels is more stable [73]. What's more, synthetic hydrogels have unique advantages in biliary stent because of their swelling properties. For example, Nagakawa et al. developed a novel self-expanding biliary stent made from PVA hydrogel in 2022. They evaluated the swelling behaviour of the stent and demonstrated the ability to dilate the bile duct effectively while maintaining unobstructed bile flow (Fig. 3B) [74]. Sodium alginate hydrogel has emerged as a promising candidate for use as ECM due to its unique properties and advantages over other biomaterials [75]. This naturally occurring polysaccharide can be obtained from brown seaweed and has several notable features, including biocompatibility, biodegradability, and high water-holding capacity [76]. For example, Alonso et al. developed a collagenous biliary stent coated with a sodium alginate hydrogel layer, which was well-tolerated after implantation, and resulted in the formation of new-born biliary epithelium at the graft site, leading to satisfactory biliary repairing outcomes [20]. Gelatin, a biological material widely used in food and drug development, is derived from denatured or partially hydrolysed natural collagen (mainly type I collagen) [77]. Compared with collagen, gelatin has lower immunogenicity, and its skeleton still maintains cell adhesion and matrix metalloproteinase (MMP)-sensitive degradation sites, making it advantageous due to its good biocompatibility, degradability, low cost, and easy preparation [78]. For example, Uemoto et al. investigated the usefulness of an artificial bile duct stent made of gelatin hydrogel nonwoven fabric (GHNF) to solve the problem of biliary reconstruction. The GHNF stent facilitated successful bile duct regeneration in rats by promoting cell migration, extracellular matrix synthesis, angiogenesis, and epithelialization [79]. Methacrylate gelatin (GelMA), prepared by chemical modification combined with methyl propylene group, has also attracted more and more attention due to its superior biological functions and mechanical properties compared to other biological hydrogels [80,81].

Hyaluronan (HA) based hydrogels can mimic the extracellular matrix and facilitate the differentiation of hepatoblasts to cholangiocytes [82]. The presence of CD44 receptors and toll-like receptor 4 in cholangiocytes suggests that HA may regulate cholangiocyte function through interactions with these receptors [83,84]. However, there is limited research on the use of hyaluronic acid in biliary stents, for the possible reason that HA may not be strong enough to resist the forces exerted by the bile duct. More researches are required to fully understand the potential benefits and limitations of hyaluronic acid for use in biliary stents.

Moreover, hydrogels can be combined with different small molecules according to specific needs, which can "intelligently" adjust the properties of hydrogels and obtain special functions to meet those needs. GelMA combined with PEGDA can improve the degree of crosslinking and mechanical strength of hydrogels and prolong the degradation process [85,86]. Additionally, GelMA can be combined with self-assembling peptides amphiphiles (PA) to form a bioink that can be used for 3D bio-printing of cell-based biology. The IKVAV-PA polypeptides can simulate the middle mucinin component of ECM, promote the growth and proliferation of cholangiocytes, and self-assemble to form functional tubular structures, which have been widely used [87]. Furthermore, mixing a contrast agent in GelMA can help researchers detect the state of the scaffold in real time through in vitro imaging instruments. For example, ultra-small superparamagnetic iron oxide (USPIO) is a novel MRI contrast agent that, when combined with GelMA and wrapped in an absorbable polymer scaffold, can make a composite biliary stent. It can be used to guide and view the repair and degradation of the biliary tract under MRI guidance [88,89]. In order to meet the requirements of various preparation methods, Guo et al. successfully synthesized norbornene-functionalized neutral soluble collagen (NorCol) [90]. It is possible for NorCol to respond to multiple stimuli, leading to an excellent candidate for bio-printing, especially in temperature-based, ion-based, and photo-based bio-printing.

## 3 Cells sources and organoids employed in creation

There are two critical elements of tissue engineering: cells sources and fabrication methods. Due to injury and inflammation, there is a paucity of primary biliary epithelial cells, which necessitates the introduction of cells for rejuvenation. A widely used approach for tissue repair is to recruit endogenous stem/progenitor cells, which guides stem progenitor cells based on chemokines, cytokines, growth factors or synthetic agents for in situ repairing [93,94]. Recruitment of endogenous stem cell has been demonstrated in the repair of musculoskeletal, cardiovascular, nervous, renal, and hepatic injuries, requiring GMP-confirming chemokine synthesis, appropriate chemokine delivery systems and pharmaceutical regulation [94]. However, endogenous stem cell recruitment in biliary repair has never been noticed in literature, possibly owing to the difficulty in recruiting iPSCs or MSCs from the circulation to the damaged location in the bile fluid environment of the bile duct. Furthermore, chemokines may undergo unanticipated release, further exacerbating of biliary inflammation, fibrosis, and stenosis.

Directly introducing epithelial cells to recover bile duct is a more often employed strategy in biliary stent research. Researchers have attempted to isolate and obtain primary cholangiocytes for mechanism exploration, cell therapy, and drug screening of biliary disease. Unfortunately, healthy bile duct donors are scarce, and primary cholangiocyte isolation is difficult and ineffective. The obtained primary cholangiocytes, however, are only capable of a single passage and are challenging to maintain in culture for an extended period of time, making them unsuitable for use in research and application. With the development of regenerative medicine and stem cell technology, researchers are primarily concentrating on the following aspects in order to produce viable alterations instead of active biliary cells sources. (1) using cholangiocyte organoids through three-dimensional (3D) culture. (2) inducing stem cells differentiated to mature cholangiocyte or cholangiocyte-like cells (CLCs). (3) selecting other cells sources for alternatives. With plantation of bile duct seeding cells, artificial biliary scaffolds for bile duct damage repairing are under deeply investigation. Comparison between different cell sources and organoids for biliary stent are listed in Table 2.

**Table 2:** Advantages and disadvantages of different cell sources and organoids for biliary stents fabrication.

Cell Source	Advantages	Limitations	Ref
Primary Cholangiocytes	Directly isolated from biliary tissue	Poor proliferation in vitro	[[95], [96], [97]]
	Maintain native phenotypes and functions in suitable environments	Difficult to maintain phenotypes in 2D culture	
		Limited availability	
Cholangiocyte Organoids	Maintain crucial functions and characteristics	Require specific culture conditions	[102,103]
	Mimic physiological processes in vitro	Loss of transcriptional diversity in organoid culture	
	Suitable for bioengineered biliary applications	Technical complexity in preparation	
	Can be derived from multiple parts of the biliary system		
iPSCs	Self-renewal and multipotential differentiation capacity	Efficiency of differentiation varies	[[108], [109], [110]]
	Can be induced to differentiate into mature cholangiocytes	Requires precise regulation of reprogramming and differentiation	
	Avoids ethical issues associated with embryonic stem cells	Potential for genetic instability	
Hepatic Progenitor Cells	Potential to differentiate into mature cholangiocytes	Immortalized lines like HepaRG have carcinogenic risks	[111]
	Can be derived from hepatocytes through dedifferentiation	Limited clinical applicability due to potential for carcinogenicity	
	Multiple differentiation pathways available		
MSCs	Widely available from various tissues	Efficiency of differentiation into cholangiocytes can be low	[124,125]
	Low immunogenicity and ability to inhibit immune rejection	Requires specific induction conditions	
	Pluripotent differentiation potential	Potential variability in differentiation outcomes	
	Can promote tissue repair and regeneration		

### 3.1 Primary cholangiocytes

Primary cholangiocytes, which are isolated from biliary tissue, are poor in proliferation in vitro and not feasible to maintain phenotypes, especially in two-dimensional (2D) culture conditions due to absence of microenvironment in vivo [95]. Du et al. isolated cholangiocyte cell line from normal mice (BALB/c) and immortalized cells by transfection with SV40.vector. They implanted immortalized primary cholangiocytes on a fabricated bile duct-on-a-chip and observed essential features of bile duct and organ-lever functions on this platform [96].

Smith et al. segregated human intrahepatic cholangiocytes (IHC) from human liver tissue and enriched IHCs for CK19. IHCs maintained cellular profiling with biliary surface markers and enzymatic activity. And branching morphogenesis of IHCs was proved to depend on extracellular matrix presentation with mitogen such as EGF, activating Notch-dependent manner [97]. Cultured in 3D environment, cholangiocytes could form spheroids with a central lumen (cholangiocyte organoids), which were pretty suitable for bioengineered biliary application.

### 3.2 Cholangiocytes organoids

The organoids are 3D cell culture products. Organoids possess certain spatial structures, which help to maintain crucial functions and characteristics of related tissues or organs and mimic the physiologic process in vitro [98]. Since organoids have unique advantages compared to 2D culture, they are broadly applied in disease model, drug screening, tissue engineering and regenerative medicine [99,100].

Multiple parts of biliary system (gallbladder, extrahepatic or intrahepatic bile duct) are all viable sources of cholangiocyte organoids. Sampaziotis et al. developed a method of human biliary epithelium separation and extrahepatic cholangiocyte organoids (ECO) forming for bile duct repairing [101]. They obtained normal cholangiocytes through dissecting extrahepatic bile duct and cultured these cells with EGF, R-spondin1 and DKK1 to turn into ECOs. ECOs could steadily express cholangiocyte-markers (CK7, CK19, SOX9, CFTR) and secrete bile. After implanting in renal capsule of NSG mice, ECOs could self-assemble into lumen structure and place on polyglycolic acid (PGA) scaffold for bile duct reconstruction [102]. In further studies, primary cholangiocytes collected from different parts of biliary tree can also be source of ECOs. Single-cell RNA sequencing results revealed that primary cholangiocytes derived from intrahepatic bile duct, common bile duct and gallbladder displayed transcriptional diversity. This transcriptional diversity might be related to different concentration of bile, which was low in intrahepatic bile duct, medium in common bile duct and high in gallbladder. In organoid culture, cells would lose this transcriptional diversity and resume in environment with bile. Meanwhile, ECOs reserved the plasticity when injected into impaired bile duct, indicating a potential of repairing different types of bile duct injury. Through ex vivo normothermic perfusion (NMP) technology, recipient liver appeared ideal repairing effect and higher production of bile [103].

The primary cholangiocytes for organoid culture could come from many approaches such as liver biopsy, bile duct biopsy and bile collection. Willemse et al. demonstrated that cholangiocyte organoids derived from extrahepatic bile duct and bile were more applicable to implant on tissue engineering biliary stents. These cholangiocyte organoids could express mature cholangiocyte markers, elevate electric impedance and enhance barrier function of biliary walls [104].

The decellularized liver scaffolds can facilitate functional cholangiocyte organoids formed. Chen et al. constructed functional ductal organoids (FDOs) with biliary networks through seeding primary cholangiocytes into decellularized liver scaffolds via retrograde infusion along biliary tract. FDOs appeared high viability, specific biomarkers and polarity, and integrated functions of bile secretion and transportation. Furthermore, FDOs expressed key molecules of the tryptophan metabolism pathway, which was associated with biliary reconstruction, during the culture [65].

The viscoelasticity of hydrogel influences the features of cholangiocyte organoids to a great extent. Rizwan et al. developed a series of defined viscoelastic hydrogels to culture primary cholangiocyte. Yes-associated protein (YAP) target genes were significantly increased in cholangiocyte organoids. Through immobilizing Jagged1 and stress relaxing hydrogel, organoids self-assembled in bile duct structures, indicating important roles of Notch activation and viscoelasticity in organogenesis [105].

### 3.3 Human induced pluripotent stem cells

Human induced pluripotent stem cells (iPSCs) are a sort of extensively studied source of stem cells with ability of self-renewal and multipotential differentiation. Utilizing the capacity of iPSCs, researchers modified the condition of inducing differentiation, which simulated the process of biliary epitheliogenesis, and generated CLCs [106].

Regulation of iPSCs reprogramming could influence the iPSCs differentiating into mature cholangiocytes. Among the process, SRY-box 17 (SOX17) plays crucial6 roles in differentiation and carcinogenesis of cholangiocytes. SOX17 mainly participates in the last stage of iPSC-induced CLCs formation and maintains the phenotypes and functions of cholangiocytes [107].

Sampaziotis et al. established a 5-step inducing method for inducing the differentiation of iPSCs into CLCs, including stages of definitive endoderm (DE), foregut progenitor (FP), hepatoblast (HB) and cholangiocyte progenitor (CP). Among the induction, the first four steps were proceeded in 2D culture, and the final step was accomplished in 3D culture using William's culture medium mixed with Matrigel and EGF. CLCs expressed critical cholangiocyte markers (CK19, CK7 and CFTR), and appeared cellular ciliogensis [108]. De Assuncao et al. described a system to produce mature cells through iPSCs-DE-HP-cholangiocytes procedures. In the ultimate stage of HP inducing to cholangiocytes, researchers applied H69 culture medium mixed with collagen I, Matrigel and TGF-β. In this 3D culture system, cholangiocytes could grow cystic or tubular branched structure, and express markers (CK19, CK7, CFTR and AE2). Transplanted into animal model with injured bile duct, the colonized cells could present homo-MHC1 and CK7 and transform into tubular shape [109].

How to enhance the efficacy of iPSC-induced differentiation into cholangiocytes is one of the hotspots under investigation. Ogawa et al. demonstrated that co-culturing HB cells with mesenchymal cells and adding HGF, EGF and TGF-β thereby activating Notch signaling pathway could efficiently promote the differentiation of iPSCs into mature and functional cholangiocytes in the process of HB differentiation. In the 3D culture system co-cultured with mesenchymal cells, iPSC-derived cholangiocytes could form epithelialized cystic or tubular structures, and express mature cholangiocyte markers (CK19, CFTR, and SCTR). These sac-like and tubular structures could exhibit epithelioid functions, including CFTR-mediated fluid secretion and forskolin-induced cAMP pathway activation to improve water-swelling. Transplanting those cells into NSG mice, the cells could express biliary markers and form tubular structures after 8 weeks, suggesting the possibility that the inducing differentiated cholangiocytes might be applied in clinical treatment of biliary diseases [110].

Discovering specific cellular surface markers and sorting out stem cells with pluripotent capacity could also increase the efficiency of differentiation into cholangiocytes. Kido et al. reported that carboxypeptidase M (CPM) could be used as a specific surface marker for the isolation of bi-directional differentiation potential liver progenitor cells (LPCs) in iPSC-induced HB cell populations, and further differentiated into mature cholangiocytes. CPM was upregulated in embryonic LPC, HB, iPSC-derived cells, and gradually downregulated during the hepatocyte maturation. Therefore, the researchers sorted CPM^+^ HB cells by flow cytometry and massively expanded in vitro. The sorted CPM^+^ cells have bi-directional potential to differentiate into mature hepatocytes and mature cholangiocytes. After using mixed collagen/Matrigel, adding R-spondin1 and WNT3a to establish a 3D culture system, culturing and inducing differentiation CPM^+^ cells into cholangiocytes for 1 week, the cells showed polarity and expressed F-actin, PKC, AQP1 on the apical membrane, and presented CD49f on the basement membrane. Meanwhile, CPM^+^ cells-induced cholangiocytes expressed higher level of CK7 and CFTR compared with iPSC-induced cholangiocytes [111].

Lgr5 is a surface biomarker of hepatic stem cells. Chen et al. isolated Lgr5^+^ cells from mouse liver with pluripotent differentiation potential into mature hepatocytes and cholangiocytes, respectively. Lgr5^+^ hepatocyte organoids obtained by in vitro 3D culture could be further induced into cholangiocytes. The mature cholangiocytes could express cholangiocyte markers (CK7, CK19, HNF1B, ASBT, and AE2), form ciliated structures, transport small molecules, and respond to Fxr receptor antagonists. Seeding cells on tissue-engineered bile ducts made of collagen-coated polyethersulfone hollow fibre membrane (PES-HFM), the cells can maintain their morphology and function of bile transporters [112].

### 3.4 Hepatic progenitor cells

Some precursor cells of bile duct in liver also possess potentials to differentiate into mature cholangiocytes. HepaRG is an immortalized hepatic progenitor cell line with a certain degree of differentiation pluripotency [113], and it is reported to differentiate to cholangiocytes in literature [108]. However, because HepaRG are derived from a patient with cholangiocarcinoma, it is risky that an uncertain possibility of carcinogenicity exists. Therefore, the immortalized hepatic progenitor cells are unsuitable for clinical treatment.

Hepatic progenitor cells could also be obtained through inducing hepatocytes dedifferentiated, and then induced to mature cholangiocytes. Buisson et al. provided a inducing method to obtain human chemically derived progenitor cells (hCdH) dedifferentiating from primary hepatocytes with HGF, CHIR and A83-01 chemicals. Then hCdH were cultured in 2D environment with 10 % FBS, 1 × ITS, nicotinamide, dexamethasone, EGF/HGF, CHIR and sodium taurocholate. After 2 weeks, the cells formed a clustered or tubular branched structure, and highly expressed the cholangiocyte-markers such as CK7, CK19, AE2, CFTR and AQP1. Moreover, hCdH-derived cholangiocytes had the cholangiocyte-specific function of secretion [21].

Huang et al. provided a strategy of in vitro chemical induced liver progenitors (CLiPs) from primary hepatocytes offering biliary cells. Functional CLiP-derived bile duct-like structure was able to transport bile. In addition, in vitro integrated CLiP-derived tubule-hepatocyte tissue presented an elevated performance of hepatic activity, and accumulation and transportation of bile [114].

### 3.5 Mesenchymal stem cells

Mesenchymal stem cells (MSCs) are a type of stem cells acting on the self-renewal of mesenchymal tissues. MSCs widely exist in bone marrow, umbilical cord, placenta and other tissues, which can selectively migrate into damaged tissues. MSCs have pluripotent differentiation potential, such as osteoblasts [115], neural cells [116], cardiomyocytes [117], hepatocytes [118], and are able to promote tissue repairing [119,120]. MSCs have low immunogenicity and could inhibit allogeneic immune rejection, and avoid the ethical issues of embryonic cells or embryonic stem cells for treatment. Thus, it has become one of the options for stem cell therapy and commonly used as seeding cells in tissue engineering. In the treatment of hepatobiliary diseases, there are currently studies using MSCs for the treatment of acute or chronic liver injury [121], liver fibrosis [122] and other diseases. In the micro-environment of liver, MSCs have the possibility to trans-differentiate into hepatocytes under the effect of cytokines such as HGF [118]. In liver cirrhosis and end-stage liver disease, MSCs could secrete anti-apoptotic (IL-6, IGFBP-2), anti-inflammatory (IL-1Ra) cytokines, which inhibit the activity of hepatic stellate cells to make immunosuppressive and anti-inflammatory responses effect for improving liver inflammation and fibrosis, and finally stimulate liver regeneration [122].

There are some studies about inducing MSCs differentiated into mature cholangiocytes. Zhang et al. utilized HGF to induce bone marrow-derived MSCs transforming into hepatic stem cells; then using HGF, EGF, SGF and dexamethasone inducing hepatic stem cells to differentiate into cholangiocytes. Morphology of cells changed into dendritic shape, and CK19 expression increased [123].

Sawitza et al. proposed the use of cholic acid (CA) and its derivatives to inducing MSCs into cholangiocytes. They used different types and concentrations of CA and its derivatives for inducing MSCs in culture and detected upregulation of hepatic stem cell markers (Afp, Hnf4α, Cyp7a1, Epcam), cholangiocyte marker (CK19), and downregulation of mesenchymal marker (desmin). It suggested that CA and its derivatives could induce differentiation of MSCs into cholangiocytes, especially with the most significant effect by lower concentration of tauroursodeoxycholic acid (TUDCA). TUDCA induced upregulation of bile acid transporter markers (Asbt、Oatp4、Bsep、Ntcp) expression, suggesting that cholangiocytes had normal bile acid transport function. Further mechanistic analysis revealed that CA and its derivatives could activate bile acid receptors Fxr and Tgr5 to initiate MSCs differentiation, while Notch, Hedgehog, TGF-β, and non-classical Wnt signalling pathways played important roles in the process of bile acid-mediated MSCs differentiation [124,125].

## 4 Fabrication methods of bioengineered biliary stents

Biliary stents are hollow tubular structures. At present, the fabricating processes of tissue-engineered biliary stents mainly include the following methods, which could be selected individually or in combination.

### 4.1 Cell seeding & self-assembling

**Fig. 4:**
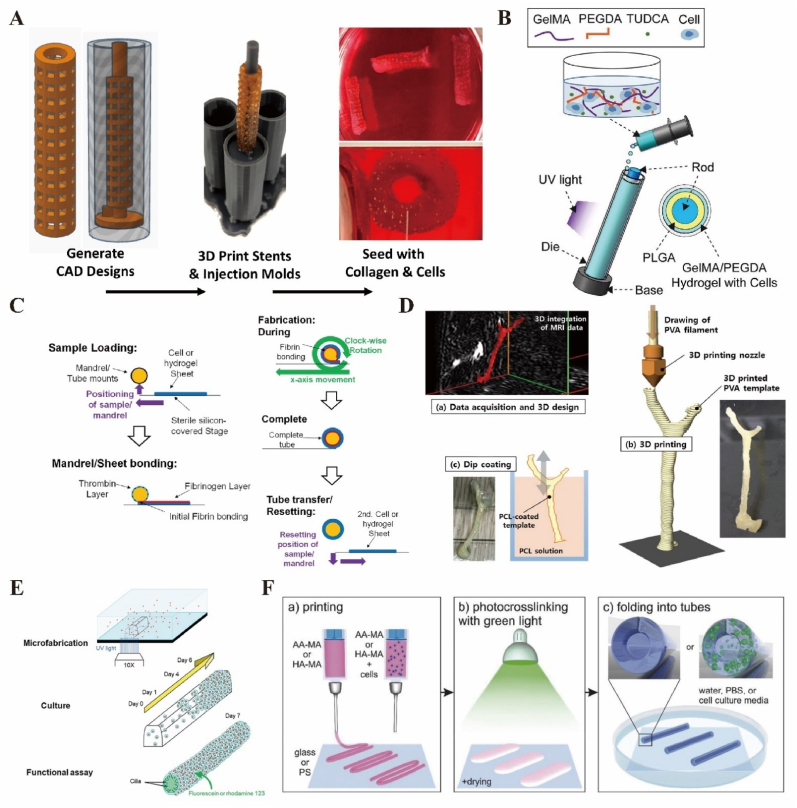
Fabrication processes of tissue engineering tubular scaffolds. (A) Scheme of PMSC collagen seeded stent [130]. Copyright © 2019 Licensee MDPI. (B) Scheme of induced-MSC-laden dual-layer tubular scaffold [91]. Copyright © 2021 Elsevier Ltd. (C) Automated fabrication strategy of patterned tubular architectures [134]. Copyright © 2015 IOP Publishing Ltd. (D) Fabrication strategy of dip coating through 3D printed template [135]. Copyright © 2017 American Chemical Society. (E) Scheme of DLP stereolithography-based biliary stent [136]. Copyright © 2021 Elsa Mazari-Arrighi et al. Published by Elsevier Ltd. (F) Scheme of the 4D biofabrication of self-folding hydrogel-based (cell-laden) tubes [137]. Copyright © 2017 WILEY-VCH Verlag GmbH & Co. KGaA, Weinheim.

Cell seeding & self-assembling refers to the process of distributing cells onto a scaffold to facilitate their attachment, growth, and proliferation to form tissue spontaneously. The cellular stents for cell seeding are commonly made of natural or synthetic polymers [126,127], decellularized animal or human donor tubular tissues [128,129] and afterwards seeded with cells, self-assembling on the surface (Fig. 4A) [130]. This preparation method has been widely used in artificial blood vessels [131], trachea [132], and bile ducts [102,112]. Cell seeding & self-assembling has several advantages. Cell seeding allows for the creation of customized tissues by selecting specific cell types to be grown on scaffolds, which can be tailored for particular therapeutic applications. By providing a suitable surface for cell attachment, cell seeding& self-assembling promotes cell growth and proliferation, which is essential for tissue repair and regeneration. Moreover, proper cell-seeding & self-assembling techniques can lead to a more homogeneous distribution of cells, which is crucial for uniform tissue development and function. However, the disadvantages of this method are that the tubular structure is relatively simple and insufficient for accurate tissue reconstruction in vivo. The cell colonization requires skilled handling and a controlled environment, which can be technically challenging. The process of seeding can sometimes cause stress of damage to cells, potentially affecting cell viability and function post-seeding and self-assembling. Meanwhile, establishing a cell seeding & self-assembling process can be time-consuming and may require expensive consumables, especially for large-scale or high-throughput applications. Scaling up cell seeding & self-assembling from laboratory to industrial levels can be difficult due to obstacles in maintain uniformity across larger volumes.

### 4.2 Injection molding

Injection molding is a versatile and cost-effective method for producing stents, from simple shapes to complex, multi-layer components. It requires careful control of mechanical parameters, mold design, material properties and processing conditions to produce high-quality parts efficiently. Using a rod core and a hollow cylindrical mold with an inner diameter larger than the diameter of rod core, the hydrogel solution laden with cells was injected into the gap between the rod core and the mold. The thickness of the stent wall was controlled by adjusting the size of the gap. After the hydrogel is cross-linked, the rod core is removed away and the hydrogel stent is taken out from the mold (Fig. 4B) [91]. In addition, biological polymer materials could be added to the structure to strengthen the mechanical strength of the tubular stent [133]. Injection molding is highly efficient for mass production, providing a high degree of consistency and repearability. Meanwhile, the design of the mold allows for high flexibility in shapes to withstand various environmental conditions. Despite its advantages, injection molding also has some drawbacks. After molding, the material can shrink as it cools, which must be accounted for in the mold design to ensure proper dimensions. Parts may develop internal stresses during the cooling process, leading to warpage or deformation. Therefore, selecting proper materials and designing more exquisite mold can help mitigate this issue.

### 4.3 Self-assembly spooling

Self-assembly spooling is a technology which combines cells spontaneous spreading and an operating system of slow-speed peripheral spooling. Cells are coated on the surface of a monolayer film structure and coated with collagen, fibrin, or ECM to form a self-assembled layer of cells, which is then shaped into a tubular structure by spool molding. Meanwhile, spooling requests multiple input/output operations, allowing the system leads to higher throughput. This method allows the thickness of the scaffold to be controlled by the layer number of the film. It is also possible to continue adding other thin film-like materials coated with cells outside the tube, thus forming a multilayer tubular structure [138]. Moreover, this method of self-assembly spooling could accomplish automatic manufacturing, offering the possibility of mass production (Fig. 4C) [134]. However, self-assembly spooling systems can add complexity to the management of input/output operations, as they require precise control to handle the queuing. Meanwhile, self-assembly spooling is not well-suited for real-time processing as it is designed for sequencing and deferred processing.

### 4.4 Dip coating

Dip coating is a method of applying a coating to a solid surface by immersing it into a liquid coating bath and the withdrawing it. In fabrication of biliary stents, a rod core is immersed in a pool of hydrogel solution containing cells for a period, and then dipped in the cross-linking agent rapidly until the scaffold is removed from the rod core to obtain a cell-laden hydrogel structure. After the coating is applied, it should solidify to form a durable film. This method could control the thickness of the scaffold wall by adjusting the standing time in the solution, or the times of repeats dipped in the same or different cell hydrogel solutions to achieve multilayer scaffolds [139]. Compared with other methods, this coating method is faster and easier to control. And dip coating is also a relatively inexpensive methods which is suitable for both small-scale application and large-scale industrial production. Furthermore, the application of coating technology can enhance the characteristics of the biliary stent surfaces, making the coatings functional and resolving complications such as infection and stenosis. The functional coatings include corrosion resisting, antibacterial, antitumour, stone-preventing, X-ray detectability and stent migration resistance, and have greatly broaden the comprehensive applications of biliary stents [140].

The disadvantages of dip coating are that it requires the preparation of a pool of hydrogel solution containing cells, therefore the material consumption is massive (Fig. 4D) [135]. Moreover, the coating may be thicker at the bottom and thinner at the top, a phenomenon known as the “wedge effect”, which is one of the main challenges with dip coating to achieve a uniform coating thickness. Besides, the coating and drying processes may require controlled environmental conditions, such as strict temperature and humidity, to ensure proper coating performance.

### 4.5 3D bio-printing

3D bio-printing has become an important approach to make bionic micro-tissues with complex structures and can also be applied in the manufacturing of tubular structural scaffolds [[141], [142], [143]]. 3D bio-printing involves the layer-by-layer deposition of biomaterials, cells, and biological molecules to create 3D biological constructs. According to different printing manners, 3D bio-printing could be mainly divided into extrusion printing, droplet inkjet printing, and digital light processing (DLP) (Fig. 4E) [136]. Co-axial printing is a widely used method to produce tubular stent. With this method, the materials are printed in a ring or spiral shape using a 3D printer, and a tubular scaffold structure is formed after printing into a certain length. One of the significant advantages of 3D bio-printing is the ability to create individual-specific construct tailored to customized anatomical and physiological needs. Although the process is time-intensive, 3D bio-printing allows for the precise placement of cells and biomaterials, enabling the creation of complex tissue structures with controlled architecture. In addition, 3D bio-printing is possible to incorporate different types of cells and materials into a construct, facilitating the creation of tissues with diverse cellular and functional compositions.

Multi-layer structure with different materials tubular stent can also be fabricated by 3D bio-printing method. Zong et al. fabricated a double-layer tissue-engineered bile duct scaffold that inner layer was made of polycaprolactone (10.13039/100018919PCL) with a dense structure and low-degradation rate to provide mechanical support; the outer layer was made of polylactic-glycolic acid (PLGA) which manufactured by 3D printing, with loose structure and high-degradation rate, and was conducive to cell growth. Seeded with MSCs, the PCL/PLGA scaffold achieved good therapeutic effects in vivo [144]. And some studies reported manufacturing 3D bio-printed absorbable biliary stent mixing with functional property, which much facilitates the clinical application [88,89]. Besides, Itoh et al. reported a printing method that arranging metal needles in a circular matrix as temporary scaffolds to place cell-containing hydrogel spheres onto the metal needles (which was also named as the Kenzan Method). After the hydrogel spheres were fused, the metal needles were removed to obtain a tubular structure scaffold [145,146].

3D bio-printing also faces some challenges and disadvantages that need to be addressed for the technology to reach its full potential. The bioink must be biocompatible, non-toxic and able to maintain the cells’ functionality post-printing; therefore, developing bioinks with right mechanical, cell-supportive and printable properties is challenging. Scaling up the production of bio-printed stent for clinical use is difficult, as it requires maintaining the quality and functionality of the bio-printed products. In addition, there are still many technical challenges to overcome, such as achieving vascularization in thicker constructs, ensuring nutrient supply to the cells, and creating tissues with mechanical properties similar to native tissues.

### 4.6 4D bio-printing

Recently, as “time” is integrated with 3D bio-printing as the fourth dimension, 4D bio-printing has been regarded as the next-generation tissue engineering [147]. This novel technology introduces the dimension of time into the process, and manufactures structures with meticulous designs for shape-morphing triggered by stimuli including internal stress [148], heat [149,150], chemicals [151], light [152] and magnetism [153]. 4D transforming constructs are usually devised from multilayer composites or programmed structural patterns. With single or multiple printable materials, printing of 4D constructs can be accomplished to achieve dynamic shape changing.

4D bio-printing has been applied for biomedical fabrication in literature. In tubular stents making, 4D-printed smart static scaffolds can respond to external stimuli and transformed into hollow shape (Fig. 4F) [137,154]. It is widely used in applications of vascular tissue [155,156], cardiac patch [157,158], neural conduit [159], intracranial stent [160] and trachea stent [161], indicating latent values in manufacturing biliary stents in future.

## 5 Applications of bioengineered biliary stents

Bioengineered biliary stents are at the forefront of medical innovation, offering advanced solutions for the management of biliary diseases. Their application encompasses various aspects of physiological and functional models, and implantation techniques.

### 5.1 Physiological and functional models of bioengineered biliary stents

Bioengineered stents are designed to mimic the physiological properties of the biliary system more closely than traditional method. Cellular bioengineered biliary stents are supposed to have expression properties of specific to cholangiocytes that match the natural bile ducts (Table 3). The cholangiocyte-specific markers, including keratin 19 (Krt19), SRY-box 9 (Sox9), and cystic fibrosis transmembrane conductance regulator (Cftr), are anticipated to demonstrate robust expression. Conversely, the levels of hepatocyte-specific markers, such as albumin (Alb), hepatocyte nuclear factor 4-alpha (Hnf4a), and cytochrome P450 family 3 subfamily a member 11 (Cyp3a11), are ideally anticipated to be minimal. Specifically, the expression of CK7 and CK19 indicate identity and maturity of cholangiocyte, as well as markers such as E-cadherin, β-catenin, and F-actin that denote cell polarity. Additionally, functional markers like CFTR, AE2, and AQP1, which are crucial for bile transport and secretion. Progenitor and stem cell markers, such as SOX9, PROM1, and EPCAM, give a comprehensive overview of the cellular characteristics and functionality of the bioengineered stents in vivo.

**Table 3:** Advantages and limitations of different types of cellular bioengineered biliary stents.

Cell types	Materials	Biomanufacturing approaches	Advantages	Limitations	Biomarkers	Ref
Primary mouse cholangiocytes or hepatoblasts	Jag1-HAO-Ln hydrogel	Self-assembly	1. Photo-triggered differentiation and spatio-temporal control; 2. Stable, mechanically tenable, compositionally defined;	1. Low accuracy; 2. Without dynamic perfusion;	1. Polarity: E-cadherin, β-catenin, F-actin; 2. Maturity: CK19, CK7;	[105,166]
Human liver ductal organoid cells	Decellularized rat liver ECM	Self-assembly	1. Potential of exponential proliferation; 2. Realization of co-recellularized culture;	Requiring sampling from the liver	KRT-19, SOX9, PROM1;	[64]
BCOs	Decellularized EHBD	Self-assembly	Personalization and convenient cell source;	1. Low accuracy and repeatability; 2. Compositionally undefined;	KRT-7, KRT-19, CFTR, SCTR;	[167]
Induced MSC-derived cholangiocyte-like cells	Inner layer: GelMA/PEGDA; Outer layer: GelMA/PEGDA;	Two-stage molding	Different mechanical properties of two layers according to different requirements	Low cytocompatibility	CK7, CK19	[92]
Induced MSC-derived cholangiocyte-like cells	Inner layer: PLGA; Outer layer: GelMA/PEGDA;	Two-stage molding	1. Biodegradable, compositionally defined; 2. Excellent mechanical properties and biocompatibility; 3. Promotion of angiogenesis;	1. Risk of biliary stent infection; 2. Low accuracy and complex preparation process;	CK7, CK19, AQP1, AE2, CFTR;	[91]
NRC	CMA-HAMA-FG-LAP	DLP stereolithography	1. Optimal microtopographic characteristics and biocompatibility; 2. Stable, reproducible, and compositionally defined; 3. Functional transport properties;	Without dynamic perfusion;	1. Polarity: ZO-1, F-actin, acetylated α-tubulin; 2. Maturity: MRP2, MDR-1;	[136]
hCdH-Chols	Inner layer: PCL fibers; Outer layer: TPU;	Extrusion-based 3D printing	1. High accuracy with reinforced mechanical durability; 2. Convenient cell source;	1. Complex preparation process; 2. Without dynamic perfusion;	CK7, CK19, CFTR, AE2, AQPR1, ZO-1; NDRG1, HNF1B, SLCs; EPCAM; PROX1, SOX4, SOX9, JAG1, HES1;	[168]
BMSCs	Inner layer: PCL; Outer layer: GelMA- USPIO;	Extrusion 3D printing	1. MRI-guided; 2. Adjustable mechanical properties;	1. Low accuracy; 2. Without dynamic perfusion;	F-actin;	[88]
primary human cholangiocytes	Synthetic: PGA; Biological: Matrigel;	Seeding	1. Optional isolation method; 2. Long-term culture;	1. Low accuracy; 2. Without dynamic perfusion;	CK 19, CK 7, Sox9 GGT, ALP, CD326 (EpCAM);	[101]
PMSCs and cholangiocytes	XL-PVA, PCL/BS, type 1 collagen	Extrusion 3D printing	1. Excellent mechanical properties; 2. Improved visibility under x-ray imaging;	1. Non-degradability; 2. Without dynamic perfusion;	–	[130]
Immortalized mouse cholangiocytes/Huh7	Sacrificial: Pluronic F-127; Template: dECM	Extrusion 3D printing	1. Realization of co-culture of cholangiocytes and hepatocytes; 2. Excellent mechanical properties and biocompatibility;	1. Without dynamic perfusion; 2. Compositionally undefined;	–	[169]
SV40 immortalized mouse cholangiocytes	PA-thiolated-gelatin	Extrusion 3D printing	1. Nanostructural features and bioactivity; 2. Excellent mechanical properties;	1. Without dynamic perfusion; 2. Complex preparation process;	CLF transportation	[87]
hMSCs	PCL/PLGA	Two-stage molding	1. Excellent mechanical properties;	1. Without dynamic perfusion; 2. Complex preparation process;	–	[144]
iPSC	Matrigel	Self-assembly	1. Convenient cell source; 2. Excellent biocompatibility;	1. Low accuracy and repeatability; 2. Compositionally undefined;	CK7, CK19, SOX9, SCTR, ASBT, AE2, AQP1, Acetyl-alpha-tubulin, CFTR;	[170]
Primary cholangiocytes	PGA/Collagen gel	Molding	1. Optional isolation method; 2. Excellent biocompatibility;	1. Low accuracy; 2. Without dynamic perfusion;	CK7, CK19, GGT, CFTR, ALP;	[102]
BMC	Polycaprolactone-polylactic acid-polyglycolic acid fibers	Seeding	1. Excellent mechanical properties and biocompatibility;	1. Low accuracy; 2. Without dynamic perfusion;	CK19	[162]

Jag1: Jagged1; HAO-Ln: hyaluronan-oxime laminin; BPU: biodegradable polyurethane; UAM: ureter acellular matrix; CFTR: cystic fibrosis transmembrane conductance regulator; EHBD: extrahepatic bile duct; BCOs: bile cholangiocyte organoids; PLGA: poly (lactic-co-glycolic acid); GelMA: gelatin methacrylate; PEGDA: poly (ethylene glycol) diacrylate; MSC: mesenchymal stem cells; NRC: normal rat cholangiocytes; CMA: type I methacrylated collagen; HAMA: methacrylated hyaluronic acid; FG: fibrinogen; LAP: lithium phenyl-2,4,6 trimethyl-benzoyl phosphinate; DLP: digital light processing; hCdHs: human chemically derived hepatic progenitor cells derived cholangiocytes; PCL: polycaprolactone; TPU: thermoplastic polyurethane; USPIO: ultrasmall superparamagnetic iron oxide; BMSCs: bone marrow derived stem cells; PGA: polyglycolic acid; XL-PVA: crosslinked polyvinyl alcohol; PMSCs: human placental mesenchymal stem cells; BS: barium sulfate; CFL: cholyl-lysyl-fluorescein; PHEA: polyhydroxyethyl-aspartamide.

To attain the physiological roles desired for cellular biliary stents, there has been a burgeoning concern in assessing the functionality of cholangiocytes, which are crucial for the stent's performance. For example, multidrug resistance protein 1 (MDR1) transporter expresses in normal biliary epithelia and play an important role in transporting substrates in biliary epithelium. Tomofuji et al. evaluated the ability of liver ductal organoids to efflux rhodamine 123, which can significantly be blocked by verapamil. Furthermore, they evaluated the function of the cystic fibrosis transmembrane conductance regulator (CFTR), which is expressed in normal biliary epithelia, using a forskolin-induced swelling assay.

Polarity is a fundamental characteristic of cells that enables them to perform their specialized functions. Cholangiocytes have a distinct apical-basal polarity, with their apical membrane facing the lumen of the bile duct and their basal membrane in contact with the underlying connective tissue. The apical membrane of cholangiocytes is rich in transporters and channels while the basal membrane of cholangiocytes is involved in the attachment and communication with the underlying extracellular matrix, which is critical for maintaining cell structure and function. Disruption of polarity in cells can lead to impaired bile flow and accumulation of toxic bile components, which can lead to cholestasis and liver damage. Overall, the polarity of cholangiocytes is essential for their specialized functions in bile secretion and transport, and disruptions in this polarity can have serious consequences for liver health. For example, Mazari-Arrighi et al. explored a promising biocompatible stereolithographic approach to encapsulate cholangiocytes into geometrically controlled 3D hydrogel structures to guide them towards the formation of branched tubular networks. F-actin distributed along cell-cell borders. ZO-1 staining suggested the formation of an intact epithelial barrier. Moreover, alpha-tubulin staining showed that cholangiocytes formed primary cilia facing the luminal space.

However, in the studies of biliary stents, the standardization of characterization techniques is crucial for comparing results across different studies. In vitro experiments often assess the expression of biliary cell markers such as CK19, CK7, and CFTR, as well as the functionality of bile secretion and transport. Additionally, animal experiments are essential for verifying biocompatibility, degradation characteristics, and functional recovery in vivo. Standardizing these characterization methods can enhance data reproducibility and reliability, making results from different studies comparable. In this way, it can advance the field and facilitate clinical translation.

### 5.2 Implantation techniques of bioengineered biliary stents

**Fig. 5:**
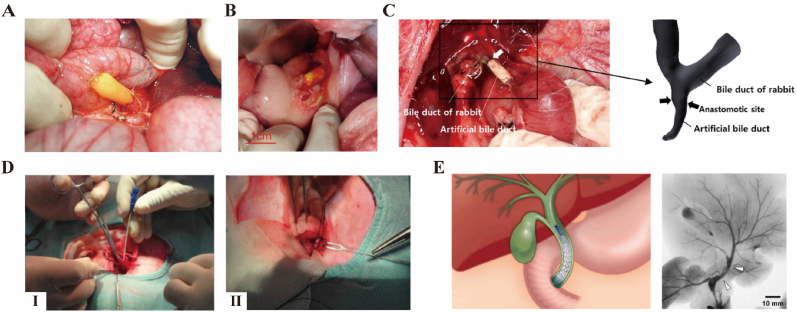
Implantation techniques of bioengineered biliary stents. (A) Anastomosis of CBD and the descending duodenumat with a biliary stent [162]. Copyright © 2005 American Society of Transplantation & American Society of Transplant Surgeons. (B) Anastomosis of biliary stent and CBD via laparotomy [91]. Copyright © 2021 Elsevier Ltd. (C) The procedure of bioengineered biliary stent implanting under light microscopy [135]. Copyright © 2017 American Chemistry Society. (D) Process for placing an advanced stent into the rabbit common bile duct through a modified central venous catheterization. Ⅰ, the guide wire and plastic jacket tube inserted into CBD; Ⅱ, the stent sutured to the inner wall of CBD [165]. Copyright © 2013, Spandidos Publications. (E) The procedure of bioengineered biliary stent implanting and releasing via balloon catheter under fluoroscopy [52]. Copyright © 2024, KeAi Communications Co. Ltd.

The deployment of bioengineered biliary stents is crucial for comprehensively evaluating their performance and ensuring their viability for clinical applications. Such stents are typically implanted in the common bile duct, which serves as their primary application site. This targeted implantation is strategic, leveraging the stent's design to address specific biliary conditions with precision. Miyazawa et al. created a series of artificial biliary stents using bioabsorbable polymers and implanted them in 18 pigs, presenting that they could replace the natural bile duct. Cholangiocyte organoid units were anastomosed to the native CBD and the descending duodenum to restore the bile duct (Fig. 5A) [162]. Similarly, Meng and colleagues developed a helical PLLA stent and tested it in a model of bile duct injury in three canines. In this model, the CBD was transected, and the stent was applied to perform a duct-to-duct anastomosis [163]. Moreover, Xu et al. conducted a study to assess the degradation characteristics and potential applicability of biodegradable PLGA stents in repairing and reconstructing the CBD. Under an operation microscope, the researchers utilized these stents in both CBD exploration and primary suturing procedures in rats [164]. Yan et al. fabricated a dual-layer cell-laden biliary stent and implanted in lesion site of for regeneration via surgical method in pigs (Fig. 5B) [91]. Park et al. achieved a thin-wall biliary stent via dip-coating method. In implantation, the artificial biliary stent was anastomosed end-to-end of the transected extrahepatic bile duct under light microscopy (Fig. 5C) [135]. Besides, interventional methods offer an optimal alternative to common bile duct (CBD) removal, allowing for the implantation of bioengineered biliary stents through minimally invasive procedures. Chen et al. used a modified central venous catheterization set as a stent introducer system to pass a biodegradable Mg-6Zn alloy CBD stent through the duodenal papilla in rabbits (Fig. 5D) [165]. Lee et al. developed a 3D-printed versatile biliary stent with nanoengineered surface. After laparotomy, an 18G angiocatheter was inserted into Vater ampulla of duodenum and a microguide wire was negotiated into CBD through the angiocatheter under fluoroscopy. Subsequently, the versatile biliary stent was placed via a balloon catheter and released following the balloon catheter fully inflated [52] (Fig. 5E). Comprehensive overviews of keys in vivo studies are presented in Table 4.

**Table 4:** Comprehensive overview of bioengineered biliary stent in vivo studies.

Animal Model	Methodology	Observed Outcomes	Ref
Pigs	Created artificial biliary stents using bioabsorbable polymers and implanted in vivo	Successfully replaced the natural bile duct	[162]
Canines	Developed helical PLLA stent and tested in bile duct injury model	Performed duct-to-duct anastomosis effectively	[163]
Pigs	Developed a dual-layery GelMA/PLGA stent	Successfully repaired injuried bile duct	[91]
Rabbits	Assessed a thin-wall PCL biliary stents in vivo	Used in CBD end-to-end anastomosis procedures	[135]
Rabbits	Used a modified central venous catheterization set to introduce biodegradable Mg-6Zn alloy CBD stent	Successfully passed the stent through the duodenal papilla	[165]
Rabbits	3D-printed versatile biliary stent with nanoengineered surface implanted in vivo	Sufficiently inhibited bacteria and hyperplasia	[52]

Despite the promising benefits of bioengineered biliary stents, such as cell-laden hydrogels and decellularized extracellular matrix (dECM) scaffolds, their clinical success has been limited. One major reason is the difficulty in replicating the complex in vivo microenvironment, leading to suboptimal cell viability and functionality. Additionally, the mechanical properties of these materials often fall short of the requirements needed to withstand the dynamic biliary environment. Immunogenic responses and potential for infection also pose significant challenges. Furthermore, the scalability and reproducibility of these bioengineered constructs remain problematic, hindering their widespread clinical adoption. Standardizing characterization techniques and improving material properties are essential steps to overcome these limitations and enhance clinical outcomes.

## 6 Legal regulations and ethical issues

The fabrication and clinical application of biliary stents involve a number of legal regulations and ethical issues that must be carefully considered to ensure patient safety, efficacy, and ethical standards. the legal regulations ensure that biliary stents are safe and effective, while ethical considerations focus on the rights and well-being of the patients who will receive them. Both are crucial for the successful and responsible use of biliary stents in medical practice.

As biliary stents are classified as medical devices, they must adhere to the regulations set forth by governing bodies, such as the U.S. Food and Drug Administration (FDA) or the European Medicines Agency (EMA). This includes obtaining necessary approvals and clearances before they can be marketed and used in clinical settings. On quality standards, biliary stents must meet specific quality standards for medical devices, which may include international standards, such as ISO 14708, for biliary stents. These standards cover design, manufacturing, and testing to ensure the stent's safety and performance. And the production of biliary stents also must follow Good Manufacturing Practices (GMP) guidelines to ensure that the devices are consistently produced and controlled according to quality standards. Meanwhile, once a biliary stent is in usage ongoing surveillance is required to monitor its performance and safety in real-world conditions. This helps to identify any potential issues that were not apparent during pre-market testing.

In accordance with the requirements of ethics, patients must be fully informed about the procedure involving biliary stents, including the benefits, risks, and alternatives. They should be able to make an informed decision about whether to proceed with the stent placement. There should be clear criteria for patient selection to ensure that biliary stent placement is appropriate for the patient's condition. This includes considering the patient's overall health, the severity of the biliary obstruction, and the likelihood of the stent improving the patient's condition. Meanwhile, healthcare providers must avoid conflicts of interest, such as promoting a particular brand of stent due to financial incentives, which could compromise patient care. Decisions about who receives a biliary stent, especially in situations where resources are limited, must be made fairly and based on medical need. Beside, in cases where a biliary stent is used as a palliative measure for patients with terminal illnesses, ethical considerations include managing patient expectations, discussing the goals of care, and providing comfort and support.

In addition, patient data collected during the fabrication and application of biliary stents must be handled in accordance with data protection regulations to ensure patient privacy. According to analysis of patients’ data, manufacturers and healthcare providers have an ethical obligation to learn from clinical outcomes and to continuously improve stent design and application techniques to enhance patient care.

## 7 Conclusions and perspective

In this review, we summarize recent progresses of biliary stent, discuss the challenges in materials, cell sources, fabrication methods, and applications, and finally provide future perspectives in this field. Moreover, bioengineered biliary stents provide an alternative solution with advantages such as good biocompatibility and degradability, as well as the potential to accelerate the regeneration of the bile tract. Therefore, the implantation of bioengineered biliary stents is expected to become an important option for treatment of biliary disorder and has promising prospects for clinical application. The future development of bioengineered biliary stent holds great promise for patients with bile duct disorders. Here are some main concerns for the future development of this field.

First, the biodegradable biliary stents, which can naturally degrade within the body after biliary disorder recovered and reduce the need for additional procedures to remove the stent, are attracting a growing interest in clinical applications. Polymers materials such as poly(l-lactic acid) and poly(lactic-co-glycolic acid) are usually being explored for their potential in biodegradable biliary stents fabrication for the nontoxicity and biocompatibility of their decomposition products. Secondly, one of the main complication following biliary stents implantation is retrograde infection, which is caused by bacteria migrated from bowels to biliary tract. The biliary stents with anti-reflux mechanisms are being expected to prevent the backflow of duodenal contents into the bile ducts consistently, which can avoid problems with biliary inflammation, cholelithiasis and obstructions. Then, the blood perfusion plays a cruel role in maintaining the biocompatibility of implanted devices. Lacking an adequate blood supply, the milieu surrounding the stent could potentially create a suboptimal environment for tissue integration, and the deficiency in nutrients might gradually impair the stent's resistance to corrosion. Enhancing the vascularization around biliary stents represents a significant frontier in the evolution of biliary stent technology. Besides, gene-editing technology, such as CRISPR/Cas9, may allow for the creation of bile duct constructs that have specific genetic modifications. These modifications could improve the function and durability of the constructs and may also help to prevent the development of bile duct disorders in the first place. By precisely editing genes associated with cell survival and bile secretion, CRISPR/Cas9 could optimize the biological performance of cell-laden stents. In addition, advanced imaging techniques, including real-time in vivo monitoring, are crucial for assessing the integration and performance of bioengineered stents over time. These imaging modalities can provide detailed insights into the stent's biocompatibility, degradation, and functional restoration, enabling researchers to fine-tune designs and materials. Incorporating these innovative strategies will help answer key unanswered questions and drive the clinical success of bioengineered biliary stents.

Moreover, ongoing clinical trials and research will continue to shape the future of biliary stents, with a focus on evaluating the safety, efficacy, and long-term outcomes of new biliary stent designs and materials. Regulatory hurdles require thorough preclinical testing and compliance with various phases of clinical trials. Safety concerns necessitate comprehensive biocompatibility and toxicity assessments. Manufacturing and scalability issues demand adherence to Good Manufacturing Practice (GMP) standards. Ethical considerations, including informed consent and patient safety, are paramount. By addressing these factors and learning from successful case studies, researchers can navigate the complex journey from preclinical studies to clinical applications, ultimately advancing medical science and improving patient outcomes.

However, one important aspect to consider is that the application of bioengineered biliary stents, while promoting bile duct repair, indeed may increase the risk of hyperplasia, which is often a result of the interaction between the stent material and the biliary duct. These materials may cause inflammatory and immune response, leading to infiltration of lymphocytes and hyperplasia of fibroblasts in biliary duct wall [45,48]. The design of the stent, including its structure, shape, and size, can also affect the risk of hyperplasia, and degradation products from biodegradable biliary stents may stimulate cell proliferation [171,172]. Besides, the release of the drug from drug-eluting stents may be uneven, or the drug itself may lead to hyperplasia [173]. Therefore, while bioengineered biliary stents carry a potential risk of inducing hyperplasia, ongoing research and development of novel materials, surfaces modifications, and advanced manufacturing techniques require both consideration of ensuring biocompatibility and reducing risk of hyperplasia [52].

Overall, these advancements aim to improve the efficacy of biliary stents, reduce complications, and enhance the quality of life for patients undergoing biliary stent procedures. The future development of bioengineered biliary stents is an exciting and rapidly evolving field that has the potential to significantly improve the lives of patients with bile duct disorders.

## CRediT authorship contribution statement

**Jianing Yan:** Writing – original draft, Visualization, Funding acquisition, Formal analysis, Data curation, Conceptualization. **Zhichao Ye:** Writing – original draft, Visualization, Data curation, Conceptualization. **Xiaofeng Wang:** Funding acquisition, Formal analysis, Data curation. **Danyang Zhong:** Writing – original draft, Data curation. **Ziyuan Wang:** Data curation. **Tingting Yan:** Data curation. **Tianyu Li:** Data curation. **Yuyang Yuan:** Data curation. **Yu Liu:** Data curation. **Yifan Wang:** Writing – review & editing, Validation, Supervision, Resources. **Xiujun Cai:** Writing – review & editing, Validation, Supervision, Project administration.

## Declaration of competing interest

All authors state no interest of this work.

## Data Availability

No data was used for the research described in the article.
